# Wenzel the Oculist

**Published:** 1843-10

**Authors:** 


*Wenzel the Oculist.*—A lady attached to the court of the empress, becoming blind, was pronounced amaurotic by the medical man called in; her malady continuing to increase, the Baron Wenzel was sent for, and he at once declared it to be cataract, and operated on it with success. So amazed was Maria Theresa at this display of Austrian surgery, that she forthwith established a special lectureship of opthalmology, and Barth was the first that filled the chair in 1773; and in 1776 he was appointed oculist to Joseph II. He was a most expert extractor, and there are still several who have witnessed his operations—the invention and use of Beer's knife (that now so generally adopted) is in a great measure due to him, for although his was longer in the blade, and somewhat broader towards the handle, yet it was upon an enlarged scale the same. The objections urged against it, of pricking the nose from the great length of its point, and not cutting itself out (as it is termed) with facility, is now obviated in that introduced by his pupil, Beer. His mode of operating was remarkable; he did not require an assistant, (and was, perhaps, the first oculist who did not), but placing the patient standing in the corner of the room near a window, he opened the lids, and fixing the eye with one hand, he passed his knife through the cornea with the other, as is now so dexterously performed by Mr. Alexander; but, different from that very distinguished oculist, he stood before his patient. It is needless to add that he was ambidexter. He died in 1818; his portrait bespeaks him a man of noble and prepossessing appearance, and his ad captandum, but engaging manner and address, added to his acknowledged talents, procured him many admirers.—*Medical Examiner*, from *Wilde's Austria.*

